# Effective killing of the human pathogen *Candida albicans* by a specific inhibitor of non-essential mitotic kinesin Kip1p

**DOI:** 10.1111/j.1365-2958.2007.05787.x

**Published:** 2007-07-01

**Authors:** Penelope R Chua, David M Roof, Yan Lee, Roman Sakowicz, David Clarke, Dan Pierce, Thoryn Stephens, Matthew Hamilton, Brad Morgan, David Morgans, Takashi Nakai, Adam Tomasi, Mary E Maxon

**Affiliations:** Cytokinetics, 280 East Grand Avenue, South San Francisco CA 94080, USA.

## Abstract

Kinesins from the bipolar (Kinesin-5) family are conserved in eukaryotic organisms and play critical roles during the earliest stages of mitosis to mediate spindle pole body separation and formation of a bipolar mitotic spindle. To date, genes encoding bipolar kinesins have been reported to be essential in all organisms studied. We report the characterization of CaKip1p, the sole member of this family in the human pathogenic yeast *Candida albicans*. *C. albicans* Kip1p appears to localize to the mitotic spindle and loss of CaKip1p function interferes with normal progression through mitosis. Inducible excision of *CaKIP1* revealed phenotypes unique to *C. albicans*, including viable homozygous *Cakip1* mutants and an aberrant spindle morphology in which multiple spindle poles accumulate in close proximity to each other. Expression of the *C. albicans* Kip1 motor domain in *Escherichia coli* produced a protein with microtubule-stimulated ATPase activity that was inhibited by an aminobenzothiazole (ABT) compound in an ATP-competitive fashion. This inhibition results in ‘rigor-like’, tight association with microtubules *in vitro*. Upon treatment of *C. albicans* cells with the ABT compound, cells were killed, and terminal phenotype analysis revealed an aberrant spindle morphology similar to that induced by loss of the *CaKIP1* gene. The ABT compound discovered is the first example of a fungal spindle inhibitor targeted to a mitotic kinesin. Our results also show that the non-essential nature and implementation of the bipolar motor in *C. albicans* differs from that seen in other organisms, and suggest that inhibitors of a non-essential mitotic kinesin may offer promise as cidal agents for antifungal drug discovery.

## Introduction

Mitosis, the process of nuclear division that produces daughter cells that are genetically identical to each other and to the parent cell, is required for cell proliferation. Inhibition of mitosis by small molecules has contributed to the discovery of fundamental principles of cell biology in model organisms (Hoyt *et al*., 1991; Li and Murray, 1991; Dorer *et al*., 2005), and the development of novel agents to treat cancer in humans (reviewed by Jordan and Wilson, 2004; Miglarese and Carlson, 2006; Warner *et al*., 2006).

The process by which chromosomes are equally distributed to dividing cells is carried out by a transient cytoskeletal structure termed the mitotic spindle. The mitotic spindle is a bipolar structure comprised of dynamic microtubule polymers along which chromosomal movements are executed. Spindle microtubules are nucleated by centrosomes (known as spindle pole bodies in fungi) in co-ordinated arrays in response to cell cycle progression cues. Of paramount importance to mitosis is the appropriately timed co-ordination of nuclear division events with cell division cycle proceedings such that chromosomes are segregated precisely in relation to events such as cytokinesis. Although tubulin is the major protein component of the mitotic spindle, many additional proteins contribute to the process, including microtubule-based motor proteins that translate chemical energy into mechanical forces that help drive the motility events of mitosis. Kinesins utilize energy derived from the hydrolysis of ATP to produce mechanical force along microtubules to effect intracellular transport of cargo or sliding of microtubules (Vale and Fletterick, 1997). Bipolar kinesins of the bimC (Kinesin-5) subfamily are critical during the earliest stages of mitosis to mediate spindle pole body (SPB) separation and formation of a bipolar mitotic spindle in eukaryotic organisms from yeast to humans (Enos and Morris, 1990; Hagan and Yanagida, 1990; Hoyt *et al*., 1992; Roof *et al*., 1992; Sawin *et al*., 1992; Heck *et al*., 1993; Blangy *et al*., 1995). Members of this family are thought to function as bipolar tetramers that localize to the spindle in a phosphorylation-dependent manner and cross-link antiparallel microtubules to establish and maintain the bipolar spindle (Sharp *et al*., 1999).

Bipolar kinesins are reported to be essential for viability of all organisms studied to date. The first bipolar kinesin, bimC, was discovered in the filamentous fungus, *Aspergillus nidulans*, in studies of nuclear division (Enos and Morris, 1990). Mutations in the *bimC* gene resulted in a mitotic arrest characterized by a mono-astral spindle, suggesting an early role for bimC in the co-ordination of the events required for SPB separation and bipolar spindle formation. In the budding yeast *Saccharomyces cerevisiae*, two bimC homologues, ScKip1p and ScCin8p, play redundant, essential roles in mitosis. Similar to that seen with *A. nidulans*, loss of bipolar kinesin function in *S. cerevisiae* results in growth arrest characterized by mononucleate, large-budded cells with duplicated SPBs that have not separated to form a bipolar spindle (Hoyt *et al*., 1992; Roof *et al*., 1992). These results show that a failure of bipolar kinesin function results in the co-ordinated interruption of both the nuclear and cell division cycles in *S. cerevisiae*, suggesting that cell cycle progression through mitosis is precisely monitored through spindle function integrity.

*Candida albicans*, the most frequently isolated human fungal pathogen, is a multimorphic commensal fungus whose ability to switch between the yeast-like and filamentous growth forms is essential for pathogenicity (Lo *et al*., 1997; Braun *et al*., 2000; 2001; Saville *et al*., 2003). In its yeast growth mode, *C. albicans* resembles *S. cerevisiae* in co-ordinated control of the nuclear division and cell division cycles; the nucleus divides after daughter cell formation and prior to cytokinesis. However, while growing in filamentous forms, the nuclear division cycle of *C. albicans* may become unlinked from the cell division cycle as observed by the formation of hyphal projections independent of the nuclear division cycle (Hazan *et al*., 2002). Understanding the roles of components required for mitosis in *C. albicans* is likely to provide insight into how mitotic events are regulated and possibly provide a foundation for antifungal drug discovery.

The genome of the pathogenic fungus *C. albicans* has been sequenced (Jones *et al*., 2004), and within it, one open reading frame (ORF) (locus tag CaO19.712) was found with homology to known bipolar kinesins. We investigated the role of *CaKIP1* in *C. albicans* viability and mitosis, and studied the effects of specific inhibition of CaKip1p *in vitro* and *in vitro*. Using an inducible gene excision technique, we show initial loss of CaKip1p included a switch to elongated growth mode and a mitotic delay marked by aberrant rounds of SPB duplication in the absence of cytokinesis. A *Cakip1* null, viable strain was ultimately recovered, indicating that unlike previously described bipolar kinesins, *CaKIP1* is not essential for viability. A recombinant *Escherichia coli*-expressed CaKip1p motor domain fragment showed microtubule-dependent ATPase activity *in vitro* that was inhibited in a dose-dependent fashion by an aminobenzothiazole (ABT) compound via a mechanism that produced a rigor-like association of the motor with microtubules. This inhibitor acts as a cidal antimitotic compound in *C. albicans*, which arrests cells in an elongated state with a novel phenotype marked by the presence of aberrant numbers of duplicated SPB pairs. Together, these data describe a novel tool molecule for inhibition of *C. albicans* mitosis, establish a role for *CaKIP1* in mitosis and suggest that a non-essential gene involved in *C. albicans* mitosis may provide a novel opportunity for antifungal drug discovery.

## Results

### One bipolar kinesin gene exists in the *C. albicans* genome

In contrast to *S. cerevisiae*, which contains two functionally redundant members of the bimC family (ScCin8p and ScKip1p), the *C. albicans* genome carries one gene encoding a protein homologous to the bimC family of bipolar kinesins [Supplementary Fig. S1, assembly 19 (http://www-sequence.stanford.edu/group/candida/)]. We designate the *C. albicans* gene *CaKIP1* because it is similar to the *ScKIP1* gene in that it lacks the segment encoding ∼100 amino acids present in *ScCIN8* but absent in other characterized kinesin-related proteins (Hoyt *et al*., 1992).

### CaKip1p localizes to the mitotic spindle

**Fig. 2:**
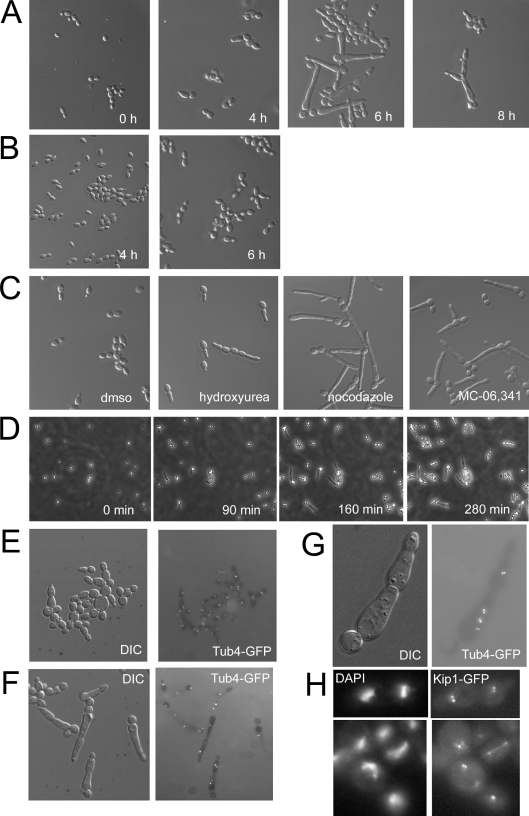
Initial loss of CaKIP1 is deleterious A. Morphology over time of cells (CKFY288) in which *CaKIP1* had been excised. Following excision in YCB-BSA, cells were back-diluted into fresh YPD medium and incubated at 30°C. Aliquots were examined at various time points under the microscope. B. Morphology of control cells (CKFY286) which contained a second copy of *CaKIP1* at the normal genomic locus. C. Morphology of wild-type cells (BWP17) treated with various concentrations of compounds. Log-phase cells to which compounds had been added were incubated for 5 h at 30°C and processed for microscopy. Compounds were added at the indicated concentrations: hydroxyurea, 300 mM; nocodazole, 25 μM; MC-06,341, 600 μM. D. Time-lapse photographs of a field of cells (CKFY288) in which *CaKIP1* had been excised. E. Visualization of spindle-pole bodies in control cells (CKFY384) 6 h after treatment in YCB-BSA. F. Visualization of spindle-pole bodies in *CaKIP1*-excised cells (CKFY373) 6 h after treatment in YCB-BSA. G. An enlargement of a cell (derived from CKFY373 6 h after excision) containing three pairs of spindle-pole bodies clustered close together within the same cell as defined by the constrictions at the bud necks separating individual cells. H. Visualization of Kip1-GFP in strain CKFY186 in which the only genomic copy of Kip1 has been fused to GFP.

To determine the localization pattern of CaKip1p, a strain in which GFP was fused to the C-terminus of *CaKIP1* was constructed. The GFP signal is concentrated to subcellular structures that resemble spindle-pole bodies (Fig. 2H, upper panels). Occasionally, a more diffuse signal is seen stretched between two concentrated GFP signals (Fig. 2H, lower panels) in a pattern that strongly resembles tubulin localization in yeast cells undergoing mitosis. Our data suggest that CaKip1p localizes to SPBs and to the mitotic spindle.

### CaKIP1p is a non-essential bipolar kinesin

To determine if cells could survive in the absence of *CaKIP1*, the construction of a homozygous gene knockout was attempted with standard gene disruption techniques (Wilson *et al*., 1999). Heterozygous knockout strains were constructed in which the entire ORF of the first copy of *CaKIP1* was replaced with the *HIS1* marker. Attempts to knock out the second copy of *CaKIP*1 by replacing it with the *ARG4* marker via direct transformation were unsuccessful. Out of approximately 200 transformants screened, no homozygous *Cakip1* knockout strains were recovered.

Further, we employed a gene excision strategy using the FLP recombinase to ultimately generate a strain lacking the *CaKIP1* gene. This approach provides not only a test of gene essentiality but also an opportunity to evaluate any terminal phenotype associated with the loss of the gene product of interest over time (Michel *et al*., 2002). Strains (e.g. CKFY302, CKFY310) were constructed in which the only copy of *CaKIP1* and the drug resistance marker *MPA*^*R*^, were flanked by *FRT* sites in a strain harbouring an integrated copy of the *FLP* recombinase gene under control of the SAP2 promoter, which is induced in the presence of bovine serum albumin (BSA). Induced expression of the FLP recombinase resulted in recombination between the *FRT* sites and subsequent excision of the *CaKIP1* gene.

**Fig. 1:**
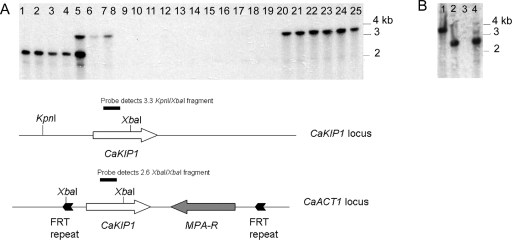
Deletion of *CaKIP1* is not lethal. Genomic DNA was digested with KpnI and XbaI and probed with radio-labelled DNA corresponding to 736–1508 of the *CaKIP1* ORF. The 3.3 kb fragment indicates the presence of *CaKIP1* sequences at the endogenous locus and the 2.6 kb fragment indicates the presence of *CaKIP*1 sequences at the *CaACT1* locus A. Excision of CaKIP1. The following lanes correspond to genomic DNA prepared from the flowing strains (only the relevant genotypes are listed here; the full genotypes are in Table 3): 1: CKFY288 (*kip1::HIS1/kip1::ARG4 ACT1/act1:: FRT-KIP1-MPA*^*R*^*-FRT*); 2: CKFY290 (*kip1::HIS1/kip1::ARG4 ACT1/act1:: FRT-KIP1-MPA*^*R*^*-FRT*); 3: CKFY302 (*kip1::HIS1/kip1::ARG4 ACT1/act1::FRT-KIP1-MPA*^*R*^*-FRT)*; 4: CKFY310 (*kip1::HIS1/kip1::ARG4 ACT1/act1::FRT-KIP1-MPA*^*R*^*-FRT)*; 5: CKFY297 (*kip1::HIS1/KIP1 ACT1/act1::FRT-KIP1-MPA*^*R*^*-FRT*); 6: CKFY35 (*kip1::HIS1/KIP1*); 7: BWP17 (*KIP1/KIP1);* 8–13: colonies derived from CKFY302 after excision; 14–19: colonies derived from CKFY310 after excision; 20–25: colonies derived from CKFY297 after excision. B. *CaKIP1* was added back to CKFY329. Lane 1: BWP17 (*KIP1/KIP1);* 2: CKFY310 (*kip1::HIS1/kip1::ARG4 ACT1/act1::FRT-KIP1-MPA*^*R*^*-FRT)*; 3: CKFY329 (*kip1::HIS1/kip1::ARG4 ACT1/act1::FRT)*; 4: CKFY741 (*kip1::HIS1/kip1::ARG4 ACT1/act1::FRT-KIP1-MPA*^*R*^*-FRT)*.

Surprisingly, FLP recombinase-induced deletion of *CaKIP1* was not lethal. This was in contrast to excision of *CDC42* from a strain carried through the process in parallel as an essential gene control for induced recombination activity. Excision of *CDC42* was reported to be first detectable 9 h after induction of FLP recombinase and was complete by 15 h within the entire culture (Michel *et al*., 2002). The kinetics of excision for *CaKIP1* should be similar given that the *CaKIP1* gene in strain CKFY302 was positioned at the same chromosomal locus as was the copy of *CDC42* in control strain SMC7A; both excised genes were flanked by identical DNA encoding the recombination sites. Following growth of the conditional *Cakip1* strain in induction media, cells were plated out on non-selective YPD media and random colonies were picked and processed with polymerase chain reaction (PCR) and by Southern blots to determine the status of the excisable *CaKIP1* gene. Of 12 colonies analysed from the CKFY302 parent strain lacking both endogenous copies of *CaKIP1*, all had lost the excisable *CaKIP1*. Six colonies from strain CKFY297 harbouring an additional copy of *CaKIP1* at the endogenous locus were also analysed and all six had also lost the excisable *CaKIP1* gene. Therefore, excision of *CaKIP1* is apparently 100% efficient under these conditions and is independent of the presence of endogenous *CaKIP1*. The viable colonies that had lost all copies of the *CaKIP1* gene could be propagated indefinitely (e.g. CKFY329). Figure 1 shows the results of the Southern blot analysis of genomic DNA prepared from the colonies described. Using a probe containing sequences complementary to the motor domain of the *CaKIP1* ORF, we demonstrate that *CaKIP1* gene sequences did not exist in the genome of the viable colonies. Given the recovery of viable strains lacking *CaKip1*, we conclude that *CaKIP1* is a non-essential gene.

**Table 3:** Yeast strains used in this study.

Strain	Genotype	Source
BWP17	*ura3::imm434/ura3::imm434 iro1::imm434/iro1::imm434* *his1::hisG/his1::hisG arg4::hisG/arg4::hisG*	Wilson *et al*. (1999)
5629	*ura3::imm434/ura3::imm434* *iro1::imm434/iro1::imm434 his1::hisG/* *his1::hisG arg4::hisG/arg4::hisG* *TUB1/TUB1::GFP-URA3*	Gerami-Nejad *et al*. (2001)
SMC7A	*cdc42–1::FRT*/*cdc42–2::FRT ACT1*/*act1::FRT-CDC42-MPA*^*R*^*-FRT* *sap2–1::*P*SAP2*_*-1*_*-ecaFLP*/*SAP2-2*	Michel *et al*. (2002)
CKFY35	*ura3::imm434/ura3::imm434 iro1::imm434/iro1::imm434 his1::hisG/his1::hisG* *arg4::hisG/arg4::hisG kip1::HIS1/KIP1*	This study
CKFY49	*ura3::imm434/ura3::imm434 iro1::imm434/iro1::imm434 his1::hisG/his1::hisG* *arg4::hisG/arg4::hisG TUB1/TUB1-GFP::URA3*	This study
CKFY171	*ura3::imm434/ura3::imm434 iro1::imm434/iro1::imm434 his1::hisG/his1::hisG* *arg4::hisG/arg4::hisG TUB4/TUB4::YFP-HIS1*	This study; derived from BWP17
CKFY186	*ura3::imm434/ura3::imm434 iro1::imm434/iro1::imm434 his1::hisG/his1::hisG* *arg4::hisG/arg4::hisG kip1::HIS1/KIP1::GFP-URA3*	This study; derived from CKFY35
CKFY286	*ura3::imm434/ura3::imm434 iro1::imm434/iro1::imm434 his1::hisG/his1::hisG* *arg4::hisG/arg4::hisG kip1::HIS1/KIP1 ACT1/act1:: FRT-KIP1-MPA*^*R*^*-FRT*	This study; derived from CKFY35
CKFY288	*ura3::imm434/ura3::imm434 iro1::imm434/iro1::imm434 his1::hisG/his1::hisG* *arg4::hisG/arg4::hisG kip1::HIS1/kip1::ARG4 ACT1/act1:: FRT-KIP1-MPA*^*R*^*-FRT*	This study; derived from CKFY286
CKFY290	*ura3::imm434/ura3::imm434 iro1::imm434/iro1::imm434 his1::hisG/his1::hisG* *arg4::hisG/arg4::hisG kip1::HIS1/kip1::ARG4 ACT1/act1:: FRT-KIP1-MPA*^*R*^*-FRT*	This study; derived from CKFY286 and independent transformant from CKFY288
CKFY297	*ura3::imm434/ura3::imm434 iro1::imm434/iro1::imm434 his1::hisG/his1::hisG* *arg4::hisG/arg4::hisG kip1::HIS1/KIP1 ACT1/act1::FRT-KIP1-MPA*^*R*^*-FRT* *sap2:: P*_*sap2*_*-ecaFLP-URA3/SAP2*	This study; derived from CKFY286
CKFY302	*ura3::imm434/ura3::imm434 iro1::imm434/iro1::imm434 his1::hisG/his1::hisG* *arg4::hisG/arg4::hisG kip1::HIS1/kip1::ARG4 ACT1/act1::FRT-KIP1-MPA*^*R*^*-FRT* *sap2:: P*_*sap2*_*-ecaFLP-URA3/SAP2*	This study; derived from CKFY288
CKFY310	*ura3::imm434/ura3::imm434 iro1::imm434/iro1::imm434 his1::hisG/his1::hisG* *arg4::hisG/arg4::hisG kip1::HIS1/kip1::ARG4 ACT1/act1::FRT-KIP1-MPA*^*R*^*-FRT* *sap2::Psap2-ecaFLP-URA3/SAP2*	This study; derived from CKFY290
CKFY329	*ura3::imm434/ura3::imm434 iro1::imm434/iro1::imm434 his1::hisG/his1::hisG* *arg4::hisG/arg4::hisG kip1::HIS1/kip1::ARG4 ACT1/act1::FRT* *sap2::Psap2-ecaFLP-URA3/SAP2*	This study; derived from CKFY310
CKFY373	*ura3::imm434/ura3::imm434 iro1::imm434/iro1::imm434 his1::hisG/his1::hisG* *arg4::hisG/arg4::hisG kip1::HIS1/kip1::ARG4 ACT1/act1::FRT-KIP1-MPA*^*R*^*-FRT* *sap2:: P*_*sap2*_*-ecaFLP-URA3/SAP2 TUB4::GFP-SAT1/TUB4*	This study; derived from CKFY302
CKFY384	*ura3::imm434/ura3::imm434 iro1::imm434/iro1::imm434 his1::hisG/his1::hisG* *arg4::hisG/arg4::hisG kip1::HIS1/KIP1 ACT1/act1::FRT-KIP1-MPA*^*R*^*-FRT* *sap2:: P*_*sap2*_*-ecaFLP-URA3/SAP2 TUB4::GFP-SAT1/TUB4*	This study; derived from CKFY297
CKFY741	*ura3::imm434/ura3::imm434 iro1::imm434/iro1::imm434 his1::hisG/his1::hisG* *arg4::hisG/arg4::hisG kip1::HIS1/kip1::ARG4 ACT1/act1::FRT-KIP1-MPA*^*R*^*-FRT* *sap2::Psap2-ecaFLP-URA3/SAP2*	This study; derived from CKFY329

### Excision of CaKIP1 is deleterious and causes a transient cell cycle perturbation

To determine any effects caused by excision of the *CaKIP1* gene, we performed a time-course analysis of cell morphology after gene excision. Following growth in induction media, cells were recovered into rich media and removed at 0, 2, 4, 6, 8 and 12 h for detailed examination. At time = 0, normal round budded cells were observed. At 2 h, the *Cakip1* cells began to take on an elongated morphology and the polarized growth continued through 6 h, resulting in extremely elongated cells with a sausage-like appearance (Fig. 2A). This phenotype was similar to what we observed in the presence of known cell-cycle inhibitors hydroxyurea, nocodazole and MC-06, 341 (Lila *et al.*, 2003) (Fig. 2C), suggesting that the initial effects of *CaKIP1* excision may result in cell-cycle arrest at least up to 6 h immediately after the loss of *CaKIP1*. At 8 h post induction, multiple new cells are observed to bud off the elongated structures, indicating that cell division has resumed.

To determine the fraction of cells undergoing elongation following *CaKIP1* excision, a time-lapse experiment was conducted. Cells from strain CKFY288 that had been grown in YCB-BSA to induce excision of *CaKIP1* were back-diluted into YPD and placed in a growth chamber heated to 30°C on a microscope stage. The cells were immobilized by placing a thin sheet of solidified YPD + agarose slab on top of them. Pictures of a chosen field of cells were taken every 10 min for 10 h. The fates of 26 cellular units were followed over time. A cellular unit refers to either single cells, or two large budded cells that were still attached together at the beginning of the experiment. 22 of the 26 cellular units were observed to grow elongated structures over the course of the experiment. Figure 2D shows representative pictures from various time points. Only cellular units that could be followed from the beginning to the end of the 10 h experiment were counted (throughout the experiment, a few cellular units continued to detach from or reattach to the surface that formed the focal plane; these units were not followed). According to the quantification, 85% of cellular units grew elongated structures characteristic of cell-cycle arrest in C. albicans. The results suggest that the majority of cells in this experiment underwent cell-cycle arrest, presumably because of the loss of *CaKIP1*. That not all cellular units elongated may be a consequence of excision under asynchronous conditions, where cells in which *CaKIP1* was excised late during the induction may have contained sufficient CaKip1p protein to avoid triggering cell-cycle arrest.

### Loss of CaKIP1 causes multiple rounds of aberrant SPB duplication

To study further the nature of the cell-cycle defect caused by initial loss of *CaKIP1*, we fused a *GFP* (green fluorescent protein) tag onto the C terminus of one copy of the *CaTUB4* gene in the conditional *Cakip1* strain, resulting in strain CKFY373. *CaTUB4* encodes gamma tubulin and when tagged with *GFP* allows visualization of the SPB. Following excision of *CaKIP1*, SPB behaviour was followed over time. Interestingly, clusters of duplicated SPBs were observed starting at 4 h following excision of *CaKIP1*. In contrast to control wild-type cells that were treated identically (Fig. 2E), about 15% of *Cakip1* cells at 6 h were marked by multiple pairs of SPBs clustered together in close proximity (Fig. 2F and G). Although these cells recover, they exhibit a slow-growth phenotype where the generation time is doubled relative to wild-type cells. Furthermore, there is an elevated proportion of elongated cells in the *Cakip1* population; during logarithmic growth in rich media, about 10% of *Cakip1* cells appear to be elongated, while roughly 1% of wild-type cells grown under identical conditions appear to be elongated.

### CaKip1p is a microtubule-dependent ATPase

**Fig. 3:**
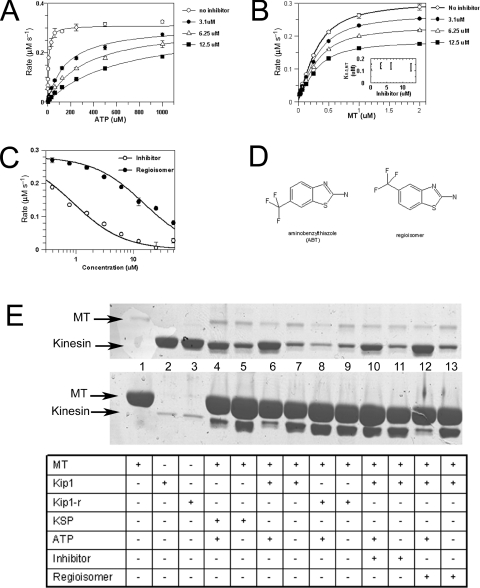
Biochemical effects of ABT on CaKip1p A–C. Steady-state kinetic analysis of CK1122684 inhibition. Each graph plots observed ATPase rate at three different inhibitor concentrations [(•) 3.1 μM, (▵) 6.25 μM and (▪) 12.5 μM] as a function of varied ATP (A) or MTs (B). Activities in the absence of inhibitor are also presented (○). Solid lines represent the best global fit to the competitive inhibition mechanism. In B, best fits to the quadratic equation are given for each inhibitor concentration. Corresponding K_0.5,MT_ values are plotted in the inset. C presents dose–response data for the effects of CK1122684 and its regioisomer on the steady state MT-stimulated ATPase of CaKip1p (obtained at 125 μM ATP and 0.6 uM polymerized tubulin). Fitted values for the concentration giving 50% inhibition by CK1122684 and its regioisomer were 0.9 ± 0.09 μM and 15 ± 1.4 μM respectively. D. Structures of the aminobenzylthiazole compound (ABT) and its regioisomer. E. CaKip1p binding to microtubules is enhanced by the presence of CK1122684. Supernatant and pellet fractions of the microtubule pelleting experiments described in *Experimental procedures* are presented in upper and lower panel respectively. Conditions of each individual experiment are indicated in the table. Human bipolar kinesin KSP was used as a positive control. Kip1-r is the rigor mutant in which the conserved glycine 297 in the Switch II region has been mutated to alanine.

The defining feature of a kinesin is its motor domain, responsible for ATP hydrolysis and motile force along the microtubule (Vale and Fletterick, 1997). We subcloned the DNA sequence encoding the conserved motor domain from *CaKIP1* and subsequently isolated bacterially expressed CaKip1p motor domain for biochemical analysis. The purified protein had a low basal ATPase rate which was accelerated over 50-fold by microtubules (Table 1), within the range expected for typical kinesin motor behaviour. We screened a collection of small synthetic organic compounds for inhibition of CaKip1p motor domain microtubule-stimulated ATPase activity and identified an ABT compound. The inhibition was ATP competitive (Fig. 3A) with a Ki of 0.14 ± 0.01 μM. ABT had no significant effect on K_0.5,MT_ indicating that the inhibition is not competitive with microtubules (Fig. 3B). A regioisomer of ABT was synthesized (where the trifluoromethyl group is in the 5 position, see Fig. 3D) and found to be significantly less active as an inhibitor of microtubule-stimulated ATPase activity (Fig. 3C), supporting the notion that ABT activity is specific for CaKip1p. We also tested ABT against other members of the bipolar kinesin family. ABT was 10- to 50-fold less active against HsKSP (human), MmKSP (mouse) and AnBimC (*A. nidulans*) motor domains but was as active against ScCin8 (*Saccaromyces cerevisiae*) as it was against CaKip1. From this motor domain inhibition survey, ABT appears to be specific for CaKip1 and its closely related budding yeast homologue, indicating that the compound is not a general ATPase or general kinesin inhibitor.

**Table 1:** Steady state kinetic constants of bacterially expressed Kip1 motor domain.

Constant	Value
Basal ATPase rate	0.018 ± 0.03 s^−1^
k_cat,MT_^a^	0.98 ± 0.3 s^−1^
K_0.5,MT_^a^	0.13 ± 0.03 μM
k_cat,ATP_^b^	1.01 ± 0.01 s^−1^
K_m,ATP_^b^	7.4 ± 0.7 μM

a Measured at 1 mM ATP, parameters fitted using a quadratic equation.

b Measured at 2 μM polymerized tubulin.

### ABT acts via a rigor state-inducing mechanism of action

During the kinetic cycle of a kinesin motor, affinity for microtubules is highly dependent on the state of nucleotide residing in the nucleotide binding site of the motor. In nucleotide free and ATP-bound states, kinesin motors are tightly attached to the microtubule lattice. In ADP and ADP-Pi states, the affinity for microtubules is much lower. A microtubule pelleting assay tests the ability of a motor protein to bind to and release from microtubules in response to the addition of ATP (Pidoux *et al*., 1996); a motor that hydrolyses ATP will release from the microtubule-bound state and partition largely in the supernatant (Fig. 3E, compare lanes 6 and 7). Knowing that the ABT inhibitor was ATP-competitive, it was of interest to determine whether ABT renders the motor in a strongly or weakly microtubule-bound state. The microtubule-binding assay indicated that in the presence of ABT, CaKip1p remains strongly attached to microtubules, forming a rigor-like complex as indicated by the decreased amount of motor protein in the supernatant and the increased amount found pelleted with the microtubules (Fig. 3E, compare lanes 6 and 10).

Motor proteins sense the presence or absence of a single phosphate group through two highly conserved loops in the catalytic core, switch I and switch II, that form hydrogen bonds with the gamma-phosphate (Vale and Milligan, 2000). Within the switch II region, a highly conserved glycine residue forms a hydrogen bond with the gamma-phosphate of the nucleotide and triggers a conformational change between the ATP and ADP states (Sablin *et al*., 1996). A mutation in this conserved glycine has been reported to block ATP hydrolysis and prevent microtubule gliding, effectively ‘locking’ the motor to the microtubule (Rice *et al*., 1999). We created the analogous mutation in the CaKip1p motor domain (G297A), expressed it in *E. coli* and tested it in the microtubule pelleting assay. This protein, CaKip1-r, bound tightly to the microtubules as expected, and was insensitive to the addition of ATP to the microtubule pelleting assay (Fig. 3E, compares lane 8 and 9). The behaviour of CaKip1p in the presence of ABT mimics permanent microtubule-binding of the ‘rigor’mutant, CaKip1-r. The effect of ABT is also similar to the effects of other known ATP-competitive kinesin inhibitors (such as non-hydrolysable ATP analogues AMPPNP and AMPPCP) on microtubule-binding [data not shown (Kapoor and Mitchison, 2001)]. The addition of the regioisomer, which does not significantly inhibit CaKip1p ATPase activity, does not cause CaKip1p to bind tightly to microtubules as does ABT (Fig. 3E, compare lanes 10 and 12). The end effect of ABT inhibition is formation of a stable CaKip1p-microtubule complex, likely to disrupt microtubule gliding. Given the requirement for microtubule dynamics during mitosis, this mechanism of action indicated that if ABT could traverse the cell membrane, it might function in a dominant-negative fashion to inhibit growth by effectively locking the mitotic spindle via rigor inhibition of a relatively small number of target molecules.

### ABT inhibits *C. albicans* growth in a CaKip1-dependent fashion

**Fig. 4:**
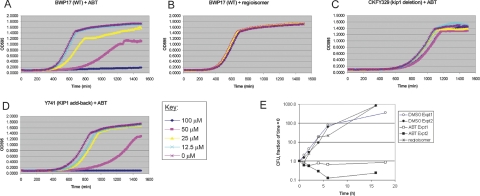
ABT inhibits *C. albicans* growth in a CaKip1p-dependent fashion. Growth curves were measured at 10 min intervals using a spectrophotometer A. Wild-type strain BWP17 was inhibited by ABT in a dose-dependent fashion. B. The wild-type strain growth was not inhibited by the regioisomer of ABT. C. *Cakip1* homozygous deletion mutant CKFY329 grew at a slightly slower rate, but showed no inhibition by ABT. D. CKFY741, derived from CKFY329 by adding back a copy of CaKIP1 at the ACT1 locus, is resensitized to ABT. E. Cell viability after exposure to 50 μM ABT declined. Data from two independent experiments are shown.

To assess the effect of ABT on growth of *C. albicans*, ABT was added to mid-log phase cells and the cell density was measured spectrophotometrically at 10 min intervals for 12 h in a kinetic growth assay. ABT caused growth inhibition in a dose-dependent fashion (Fig. 4A). A control culture treated with the same volume of dimethyl sulphoxide (DMSO) solvent exhibited exponential growth and a doubling time of 59 min. The concentration of ABT necessary to inhibit the cell density during exponential growth by 50% (the growth IC50) was 60 μM. The continued increase in optical density with time could be due to continued growth by cell elongation (rather than cell division) during the mitotic arrest induced by compound given the results observed using ABT in microscopy experiments. To measure the impact of ABT on cell viability, we performed a time-kill analysis. During the first 6 h of exposure, 100 μM ABT caused the viable cell count to decline by two- to eightfold (Fig. 4F), although some regrowth occurred during the final 10 h of incubation.

Various approaches were used to assess whether the growth-inhibitory activity of ABT resulted specifically from inhibition of the CaKip1p enzyme in cells. We measured the effect of ABT on the homozygous *Cakip1* deletion mutant (strain CKFY329) and compared the results to those seen when ABT was applied to wild-type cells. The homozygous *Cakip1* deletion mutant grew with a doubling time of 148 min and exhibited no significant inhibition by ABT at concentrations up to 100 μM, indicating that the cellular effect of ABT requires the presence of the CaKip1p target (Fig. 4C). To control for the possibility that the slow-growing homozygous deletion strain had acquired additional mutations that resulted in resistance to ABT, *CaKIP1* was added back to CKFY329 at the *ACT1* locus (Fig. 1B). The addition of *CaKIP1* back into the homozygous *Cakip1* deletion mutant resensitized it to ABT (Fig. 4D), ruling out the possibility that secondary mutations resulting from the effects of deleting *CaKIP1* had caused the strain to become resistant to ABT. A regioisomer of ABT, compound CK1122735, neither significantly inhibited CaKip1p ATPase activity nor inhibited growth of wild-type cells (Fig. 4B and E) These observations taken together strongly argue that inhibition of the mitotic kinesin CaKip1p by ABT is the cause of cidality.

### ABT blocks mitotic spindle elongation

**Fig. 5:**
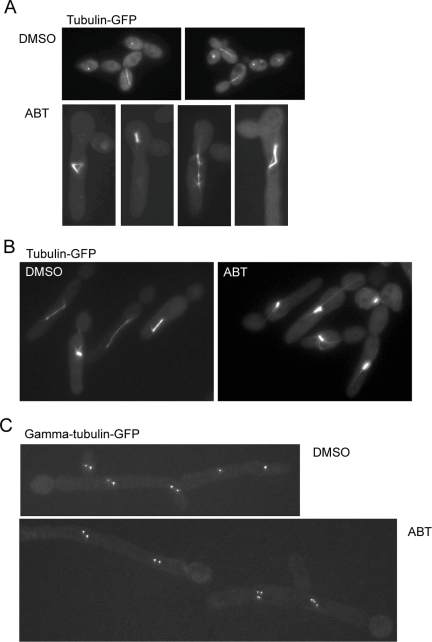
Effect of ABT on yeast cells. A. Tubulin-GFP was used to visualize spindles in asynchronously growing cells treated with 50 μM ABT or with DMSO for 4.5 h. ABT treatment resulted in cell elongation, aberrant multisegmented spindles and the absence of elongated anaphase spindles B. Cells were treated with hydroxyurea to arrest cells with short bipolar mitotic spindles, then released into medium with DMSO or 50 μM ABT and incubated for 1.5 h. C. Tub4-GFP (gamma tubulin) was used to visualize spindle poles in cells synchronized with hydroxyurea. Release into medium with 50 μM ABT for 3 h often resulted in four or more spindle poles in close proximity, while spindle poles in the DMSO control were present singly or in pairs.

A prediction of the experiments described above is that ABT should arrest cells in mitosis. To test this prediction, we performed microscopy on ABT-treated asynchronously growing cells. Treatment with 50 μM ABT for 4.5 h resulted in 63% of the cells with an elongated cell morphology (*n* = 87) similar to that seen during *CaKIP1* gene excision or treatment with cell cycle inhibitors hydroxyurea, nocodazole or MC-06,341, while no elongated cells in were observed in the control population (*n* = 70). Microtubules were observed in ABT-treated cells using GFP-tagged tubulin to assess whether the nuclear division cycle was affected, and the percentage of cells in each stage of mitosis was scored. The DMSO control culture contained cells with monopolar spindles (46%), bipolar preanaphase spindles (27%) and anaphase spindles (27%), reflecting normal cell cycle progression (Fig. 5A). In the ABT-treated culture, perturbations of mitosis were evident from the altered proportions of cells present in each stage of spindle morphogenesis and from the presence of abnormal spindle structures. Abnormal, multisegmented spindles were present in 22% of the cells (Fig. 5A). The length of each segment was similar to the length of bipolar preanaphase mitotic spindles in control cells, however, in the ABT-treated cells the segments were frequently interconnected. These structures were reminiscent of microtubules nucleated from four SPBs in close proximity to each other as observed after *CaKIP1* gene excision. In addition to the abnormal spindle structures, cells were present with spindle structures typical of monopolar (57%) and preanaphase bipolar (17%) and anaphase (4%) spindles. The reduced fraction of cells with preanaphase bipolar and elongated anaphase spindles compared with the control culture suggests that ABT interferes with bipolar spindle assembly and elongation and is consistent with the appearance of the abnormal multisegmented spindles. However, this experiment could not distinguish whether the preanaphase and anaphase spindles observed were formed in the presence of ABT, or whether these spindles were pre-existing at the time of drug addition.

To assess the effect of ABT inhibition of CaKip1p on maintenance of spindle bipolarity and elongation, we used hydroxyurea to produce a nearly uniform population of cells with an elongated bud and a bipolar preanaphase mitotic spindle. Cells were released from hydroxyurea arrest in the presence or absence of ABT, and the fate of the spindle was observed. After 30 min, both the ABT-treated and the DMSO treated cultures contained greater than 80% short bipolar spindles (Table 2), with no evidence of inward collapse of the two spindle poles to form a monopolar spindle as occurs upon loss of bipolar kinesin function in *S. cerevisiae* (Saunders and Hoyt, 1992). After 90 min, 69% of the cells in the DMSO culture had entered anaphase, while the spindles in the ABT-treated cells remained as short bipolar spindles with no anaphase structures detected in the population (Fig. 5B). After 3 h of ABT treatment, anaphase spindles were still not observed (< 2%).

**Table 2:** Spindle morphology after treatment of synchronized cells with ABT.

Treatment^a^	Time after compound addition (h)	Short bipolar	Single pole	Two adjacent poles	Anaphase	Post anaphase
DMSO	0.5	89	10	0	1	0
ABT	0.5	83	14	3	0	0
DMSO	1.5	25	3	3	43	26
ABT	1.5	85	3	11	0	0

a Synchronized cells (strain CKFY49, tubulin-GFP), were incubated in medium containing 1% DMSO or 1% DMSO plus 50 μM CK684 for the indicated time.

To visualize the SPBs after ABT treatment, the gamma-tubulin-GFP strain was synchronized with hydroxyurea and released into ABT for 3 h. Clusters of four or more SPBs in close proximity were present (Fig. 5C), consistent with the appearance of clusters of spindle poles shortly after *CaKIP1* excision. The absence of extensive SPB separation in the presence of ABT suggests that stable CaKip1p-microtubule complexes, similar to those induced by ABT *in vitro*, prevent microtubules from sliding past each other during the mitotic spindle morphogenesis, ultimately resulting in induction of cell death.

## Discussion

Precise control of mitotic events assures that chromosomes distribute appropriately into dividing cells. In studies enabled by the inducible deletion of the gene encoding the single bipolar kinesin in *C. albicans,* we note nuclear division phenotypes similar to those described for mutations in orthologues in other organisms, as well as unique aspects of the phenotype that suggest differences in the control of mitosis in this species as compared with a closely related fungal species, *S. cerevisiae*. Most notably, we show that initial cell cycle-related phenotypes caused by *CaKIP1* deletion are effectively compensated for over time, and that unlike in *S. cerevisiae*, bipolar kinesin function is not required for viability in *C. albicans*. In addition, we identified a small molecule inhibitor of a recombinant protein encoded by the *CaKIP1* motor domain and present evidence that this molecule has a cidal effect caused by induction of a dominant negative complex between CaKip1p and cellular tubulin.

Excision of the *CaKIP1* gene initially caused cell elongation, similar to morphological effects of inhibitors of both S and M phases of the cell cycle. In addition, initial responses to *CaKIP1* loss also included a unique SPB phenotype where multiple rounds of SPB duplication occurred in the absence of cell division.

Loss of bipolar kinesin activity in *S. cerevisiae* results in mitotic effects described as a uniform large-budded arrest marked by duplicated but unseparated SPBs organizing a short monopolar spindle. Three pieces of evidence suggest that *C. albicans* might control mitosis in unique ways compared with *S. cerevisiae*. First, our ability to isolate a null, viable strain lacking *CaKIP1* suggests that bipolar kinesin activity is not essential in this organism. Second, that the loss or inhibition of the *CaKIP1* gene product promotes multiple rounds of SPB duplication in the absence of cell division suggests that SPB duplication in *C. albicans* might not be subject to the same controls as that demonstrated in other organisms such as *S. cerevisiae*. Lastly, that inhibition or loss of function of the *C. albicans* bipolar kinesin results in short bipolar spindles instead of a monopolar spindle phenotype seen in *S. cerevisiae*, *A. nidulans, Schizosaccharomyces pombe* and *Drosophila melanogaster* argues that the *C. albicans* bipolar kinesin may function differently in the establishment of the mitotic spindle. SPB pairs appear to be separated and capable of organizing short bipolar spindles in the absence of CaKip1p function, and this may indicate that bipolar kinesin function is not required for this initial step in spindle assembly as previously reported (for a review see Jaspersen and Winey, 2004).

Although bipolar kinesin function may differ between *C. albicans* and *S. cerevisiae*, some of these phenotypic effects associated with bipolar kinesin loss of function, such as multiastral spindles and multiple rounds of SPB duplication, are not unique to *C. albicans*. For example, injection of mRNA encoding a rigor mutation of the bipolar kinesin from sea urchin resulted in the formation of multiastral and multinucleated cells with short bipolar spindles (Touitou *et al*., 2001), suggesting that perturbations in bipolar kinesin function may contribute to the formation of multiple non-productive bipolar spindles in multiple organisms. In addition, growth at the restrictive temperature of cells carrying the *bimC4* mutation in *A. nidulans* resulted in an additional round of SPB duplication and polyploid nuclei (Enos and Morris, 1990). These data suggest that control of mitosis in *C. albicans* may be more similar to that of more complex organisms such as the filamentous fungus *A. nidulans* and sea urchin.

A recent report regarding depletion of a polo-like kinase (CaCdc5p) in *C. albicans* proposed that defects in spindle elongation in *C. albicans* and the corresponding generation of filaments in *CaCDC5*-repressed and hydroxyurea-exposed cells suggest a link between spindle function and activation of hyphal growth (Bachewich *et al*., 2003). Here, we show that inhibitors of mitosis (nocodazole, ABT, MC-06, 341), like the S-phase inhibitor hydroxyurea, result in an elongated cellular morphology. Although we did not attempt to confirm whether the elongated cells observed with excision of *CaKIP1* or inhibition of CaKip1p by ABT were in fact hyphal, the results demonstrate that perturbation of several different events in cell cycle progression alter cell morphology similarly in *C. albicans*.

The observation of many chromosomal peculiarities of *C. albicans* perhaps foreshadowed the notion that checkpoint control of at least spindle assembly may be absent or at least different as compared with other organisms. For example, aneuploidy and trisomy of chromosomes 1 and 2 have been reported in clinical isolates, and the generation of viable strains lacking one copy of chromosome 5 after growth on sorbose medium is also well known *C. albicans* (Whelan and Magee, 1981; Chibana *et al*., 2000). Homologues of known mitotic checkpoint genes such as *MAD1-3, BUB1-3* and *MPS1* are present in the *C. albicans* genome. Moreover, a recent account of a proposed role for *MAD2* in *C. albicans* in mitotic checkpoint control proposes that a spindle assembly checkpoint exists in *C. albicans*, although it may function somewhat differently than that of other organisms (Bai *et al*., 2002). It is tempting to speculate that a deviation from strict mitotic checkpoint control might offer a competitive advantage in pathogenesis of *C. albicans* that is not required in non-pathogenic organisms such as *S. cerevisiae*.

The non-essentiality of *CaKIP1* is surprising in light of many reports of the essentiality of bipolar kinesins in nearly all organisms studied to date, but perhaps can be explained by compensatory motor activity. The formation of a bipolar mitotic spindle requires a balance of opposing forces controlled by motors that function as ‘plus’ and ‘minus’ end activities with respect to the polarity of the microtubule (for a review see Heald, 2000). Bipolar kinesins of the Kinesin-5 class are ‘plus-end’ motors, exerting force towards the ‘plus’ end of microtubules while members of the Kinesin-13 class are ‘minus-end’ motors. Initial studies in *S. cerevisiae* revealed that multiple motor activities exert antagonistic forces to ultimately control spindle pole separation, spindle assembly and length of the spindle (Saunders *et al*., 1997), and these findings have subsequently been confirmed to exist in *A. nidulans*, *D. melanogaster*, *Xenopus laevus* and mammalian cells (O'Connell *et al*., 1993; Walczak *et al*., 1998; Mountain *et al*., 1999; Sharp *et al*., 2000). Loss of bipolar kinesin function (‘outward’ force) typically causes an inward collapse of the mitotic spindle, forming a monopolar spindle, which can be counter-balanced by inactivation of an antagonistic ‘inward’ motor. This model is supported by studies in *S. cerevisiae, D. melanogaster, A. nidulans* and murine oocytes where deletion or loss of bipolar kinesin function can be suppressed by inactivation or deletion of an antagonistic motor of the Kinesin-13 class. It is possible that our method of achieving *CaKIP1* gene loss by induction of the FLP recombinase and gene excision over several hours allows the cell an opportunity to compensate for loss of CaKip1p activity by downregulating or inactivating the Kinesin-13 gene *CaKAR3* in a similar manner.

Cellular effects of a biochemical inhibitor of CaKip1p appeared to phenocopy the effects of excision of *CaKIP1* with respect to cell elongation and multiple rounds of aberrant SPB duplication. The specificity of ABT for the CaKip1p target is supported by two pieces of data: a regioisomer of ABT does not inhibit the motor or cell growth and ABT does not inhibit a strain that lacks *CaKIP1* but does inhibit the knock-in (Fig. 4). A significant difference in the effect of the biochemical target bound to its inhibitor as compared with mutational loss of CaKip1 is demonstrated in the observation that cells exposed to ABT died whereas cells initially enfeebled by loss of *CaKIP1* eventually recovered to generate a viable null strain. Biochemical experiments support the possibility that a dominant-negative activity associated with a rigor-type mechanism of inhibition may lock microtubule-motor complexes in a non-functional state that cannot easily be overcome. We propose that the cidality of this mode of action may be enhanced by the possibility that few bipolar kinesin molecules may need to be inhibited in the cell to achieve total dysfunction of the spindle.

The use of specific small molecule probes that inactivate the functions of their targets has been extremely valuable in studies of cellular processes. Tubulin was discovered through the use of the small molecule colchicine (Borisy and Taylor, 1967; Shelanski and Taylor, 1967). Inhibition of the bipolar kinesin, Eg5, with monastrol has been demonstrated in human cells and results in the formation of a monopolar spindle (Mayer *et al*., 1999). Monastrol prevents the formation of bipolar spindles in *Xenopus* egg extracts (Kapoor *et al*., 2000) but does not do so by a rigor-type mechanism of inhibition that characterizes the ABT-mediated inhibition of CaKip1p (Kapoor and Mitchison, 2001). Like the ABT-mediated inhibition of CaKip1p, monastrol also inhibits the microtubule-stimulated ATPase activity of the motor domain; however, monastrol is not competitive with ATP and functions as an allosteric inhibitor of Eg5 that blocks microtubule-stimulated ADP release (Maliga *et al*., 2002). Recent studies that interrogated the action of monastrol-mediated inhibition of Eg5 (Luo *et al*., 2004)in the presence and absence of microtubules suggest that monastrol binds to the Eg5-ADP complex, forming a Eg5-ADP-monastrol ternary complex, which can not bind to microtubules productively. It is possible that action of monastrol causes Eg5 to release from microtubules, resulting in spindle collapse (monopolar spindle), whereas ABT causes CaKip1p to lock onto microtubules, blocking microtubule sliding and spindle collapse (short bipolar spindle). In any case, the discovery of ABT is expected to provide a useful tool for further exploration of the processes that govern mitosis in *C. albicans*.

Our findings with the inhibition of CaKip1p may have significant implications for anti-infective drug discovery. Traditionally, targets considered ideal for anti-infective drug discovery are those that are essential for viability because inhibition of essential gene function is presumed to result in growth inhibition (Moir *et al*., 1999; Wills *et al*., 2000; De Backer and Van Dijck, 2003; Walsh, 2003). Our studies indicate that inhibitors of a non-essential target can also demonstrate cidal activity in a relevant fungal pathogen. Whether this is a unique case or can be expanded to include activity against additional fungal species remains to be determined.

## Experimental procedures

### Plasmids

CKFB139 contains the *CaKIP1* motor domain cloned into the pET23d backbone. A PCR fragment containing the *CaKIP1* motor domain was obtained by amplification of BWP17 genomic DNA with primers CKF056 (5′CGTACCATGGCGTCAAATATCCAAGTTGTTGTT-3′) and CKF057 (5′CCGCTCGAGTTCTGAATCATGGCCAATCAT-3′). Following digestion with NcoI and XhoI, the PCR product was inserted between the NcoI and XhoI sites in pET23-d. The three non-conventional CTG codons within the *CaKIP1* motor domain were mutated to TCG using the QuickChange Site-Directed Mutagenesis kit (Stratagene) according to instructions from the manufacturer, resulting in CKFB139.

CKFB369 contains the *CaKIP1* motor domain carrying a rigor mutation (Kip1-r) in the pET23d backbone. It was constructed by mutating the conserved glycine 297 in the Switch II region of the motor domain in CKFB139 to an alanine, using primers CK1917 (5′-CGAAAATGAATTTAGTTGATTTGGCAGCTTCAGAAAATATTAGTCGGTCAGGATCTATTG-3′) and CK1918 (5′CAATAGATCCTGACCGACTAATATTTTCTGAAGCTGCCAAATCAACTAAATTCATTTTCG-3′) according to instructions in the QuickChange Site-Directed Mutagenesis kit (Stratagene).

CKFB400 contains the full-length *CaKIP1* gene with about 1 kb (kilobase pair) of flanking sequences on either side cloned into pAFI3 (Michel *et al*., 2002). Primers CK2564 (5′-GGGGCTGCAGTCAATTGATTTAAAGGTCGTGCACG-3′) and CK2652 (5′-GGGGCTGCAGATCATCGTTGATTCTATTAGGTTGC-3′) was used to amplify a 4.3 kb fragment from BWP17 genomic DNA. The PCR fragment was digested with PstI and then inserted into the PstI site of pAF13. The resulting plasmid was digested with NotI and XhoI prior to transformation into yeast.

Plasmid CKFB514 contains both the *GFP* gene and the *SAT1* nourseothricin resistance marker cloned into the MCS of pCR2.1 (Invitrogen). The resulting insert is essentially identical to the cassettes described by Gerami-Nejad *et al*. (2001) with the exception that the selectable marker is *SAT1. GFP* was obtained from pGFP-URA3 (Gerami-Nejad *et al*., 2001) and *SAT1* was obtained from plasmid pA83 (Reuss *et al*., 2004). This plasmid was used to construct GFP fusions by direct transformation and selection with nourseothricin as previously described (Gerami-Nejad *et al*., 2001).

All relevant DNA sequences in final constructs were verified by sequencing.

### Strain construction

All strains were constructed in the BWP17 background and are listed in Table 3. *CaKIP1* disruption cassettes containing either the *HIS1* or *ARG4* marker were constructed that contained 60 bp (base pairs) of sequences flanking the ORF as previously described (Wilson *et al*., 1999). *GFP* sequences were fused in frame to the C-termini of target genes as previously described (Gerami-Nejad *et al*., 2001). The inducible knockout strain was constructed using CKFB400 and pSFL213 (Michel *et al*., 2002). CKFY35, containing a heterozygous deletion of *CaKIP1*, was transformed with plasmid CKFB400 to introduce a third copy of *CaKIP1* at the *ACT1* locus. The second copy of *CaKIP1* at the endogenous locus was then deleted by replacement with the *ARG4* marker. The inducible *ecaFLP* gene was then introduced into the resultant strain by transformation with pSFL213 that had been cut with XbaI and SacI. The resulting strain, CKFY302, is the inducible *CaKIP1* knockout strain. Subsequent induction of *ecaFLP* resulted in strain CKFY329, the *Cakip1* knockout strain in which the copy of *KIP1* at the *ACT1* locus had been excised. CKFY741, the *CaKIP1* add-back strain, was constructed by re-integrating the *CaKIP1* gene at the *ACT1* locus using plasmid CKFB400 and selecting for MPA-resistant colonies. Standard transformation techniques were used to introduce transforming DNA into yeast cells. All strains were verified by PCR or Southern blotting or both.

For verifying excision of *CaKIP1*, Southern blot analysis was carried out on genomic DNA digested with KpnI and XbaI and probed with DNA corresponding to nucleotides 736–1508 of the *CaKIP1 ORF*. The probe was made by PCR amplification using primers CK1233 (5′-CTAGTAGTACCAACTTAAATGAAAC-3′)and CK1178 (5′-GGAAACTAAATATCATAAAGCA-3′). Endogenous *CaKIP1* is identified by a 3.3 kb KpnI/XbaI fragment and *CaKIP1* integrated at the *ACT1* locus is identified by a 2.6 kb XbaI/XbaI fragment.

### Growth conditions

Yeast were grown at 30°C in YPD (supplemented with 100 μg ml^−1^ of uridine), synthetic complete medium or synthetic complete medium lacking specific nutrients. Mycophenolic acid (MPA; Sigma) was added to synthetic medium at 10 μg ml^−1^. Nourseothricin was obtained as clonNAT from WERNER BioAgents and used at a concentration of 200 μg ml^−1^ in YPD plates. Excision of *CaKIP1* was achieved by growth in YCB-BSA medium as described (Michel *et al*., 2002).

Growth curves were performed using 100 μl cultures in sealed 96 well microtitre plates, which were incubated at 30°C and the absorbance at 595 nm measured in a Tecan Genios plate reader, as described (Giaever *et al*., 2002). To measure cell viability after compound exposure, strain BWP17 was grown in YPD medium at 30°C to a concentration of 2.5–5 × 105 cells per ml, then compound was added to obtain a final concentration of 50 μM compound and 1% DMSO, and incubation was continued. At the indicated times, cells were removed, diluted and plated on solid YPD medium to score the number of colony forming units. The final dilution of cells and compound on the solid medium was 10 000-fold or greater. Greater than 100 colonies were counted for each dilution.

Microscopy to observe spindle morphology was performed using strain CKFY49 or CKFY171. For synchronization, hydroxyurea was added to a final concentration of 0.1 M and the cells were incubated with agitation at 30°C until elongated bud morphology was evident (2–3 h), the cells were washed in fresh YPD, then resuspended in YPD containing 1% DMSO as solvent. Cells were photographed in 5–10 focal planes using a Leica DMIRE microscope with motorized focus and Metamorph software. Spindle structure was scored by examining multiple z-sections and greater than 80 cells were scored for each condition.

For timelapse microscopy, cells (following growth in YCB-BSA to induce excision) were plated on the surface of a 0.17 mm Delta T dish (Fisher Scientific) coated with poly lysine, immobilized underneath a thin slab of 1% agar and overlayed with just enough liquid YPD medium to barely cover. The entire dish was placed in a heated chamber maintained at 37°C. Phase contrast images were taken every 10 min for 12 h and processed using Metamorph software.

### Biochemical assays

A construct encoding for aminoacids 1–398 of CaKip1p and C-terminal hexahistidine tag in pET23d vector was expressed in *E. coli* BL21 (DE3) strain. Protein was purified by Ni-NTA affinity chromatography followed by SP-sepharose chromatography. This procedure yielded 60 mg of soluble protein from a litre of bacterial culture. A mutated form of this protein with Switch-II substitution known (in other kinesins) to abolish ATPase activity and induce rigor-like binding to MTs was also created, expressed and purified using identical procedure. The mutated protein (Kip1-r) yield was 25 mg from a litre of bacterial culture. Kip1-r protein was soluble but had no detectable ATPase activity.

All kinetic and binding assays were conducted in PEM25 (25 mM Pipes/KOH, 2 mM MgCl2, 1 mM EGTA, 1 mM DTT) supplemented with KCl and paclitaxel as indicated.

ATPase measurements were monitored by a coupled pyruvate kinase/lactate dehydrogenase enzymatic detection system. Reaction progress was monitored by change in absorbance at 340 nm. All measurements were performed in 96-well microtitre plates in SpectraMax340 (Molecular Devices) ABT 2-amino-6-(trifluoromethylbenzothiazole) was purchased from Matrix Scientific, England, product # 1364.

Microtubule binding assays used porcine brain tubulin purified by two rounds of polymerization/depolymerization and final phosphocellulose chromatography. PEM25 buffer was supplemented with 10 μM paclitaxel and 75 mM KCl. Kip1 protein(3 μM) was mixed with 6 μM polymerized tubulin and 1 mM ATP (weak binding) or 1 U ml^−1^ apyrase (rigor binding). Kip1-r protein and human bipolar kinesin KSP were used as controls (3 μM each). Mixtures were centrifuged at 100 000 *g* for 15 min. Supernatant and pellet fractions were analysed by SDS-PAGE.

## Supplementary material

The following supplementary material is available for this article:

Fig. S1 *CaKIP1* encodes a non-essential member of the bimC kinesin family. Multiple sequence alignment of the bimC minimal motor domain from various species. Af = *Aspergillus fumigatus*; An = *Aspergillus nidulans*; Ca = *Candida albicans*; Cg = *Candida glabrata*; Sc= *Saccharomyces cerevisiae*; Hs = *Homo sapiens*; Sp= *Schizosaccharomyces pombe*. The conserved glycine required for ATP hydrolysis is underlined.

This material is available as part of the online article from:

http://www.blackwell-synergy.com/doi/abs/10.1111/j.1365-2958.2007.05787.x (This link will take you to the article abstract).

## References

[b1] Bachewich C, Thomas DY, Whiteway M (2003). Depletion of a polo-like kinase in *Candida albicans* activates cyclase-dependent hyphal-like growth. Mol Biol Cell.

[b2] Bai C, Ramanan N, Wang YM, Wang Y (2002). Spindle assembly checkpoint component CaMad2p is indispensable for *Candida albicans* survival and virulence in mice. Mol Microbiol.

[b3] Blangy A, Lane HA, d'Herin P, Harper M, Kress M, Nigg EA (1995). Phosphorylation by p34cdc2 regulates spindle association of human Eg5, a kinesin-related motor essential for bipolar spindle formation *in vivo*. Cell.

[b4] Borisy GG, Taylor EW (1967). The mechanism of action of colchicine. Colchicine binding to sea urchin eggs and the mitotic apparatus. J Cell Biol.

[b5] Braun BR, Head WS, Wang MX, Johnson AD (2000). Identification and characterization of TUP1-regulated genes in *Candida albicans*. Genetics.

[b6] Braun BR, Kadosh D, Johnson AD (2001). NRG1, a repressor of filamentous growth in C.albicans, is downregulated during filament induction. EMBO J.

[b7] Chibana H, Beckerman JL, Magee PT (2000). Fine-resolution physical mapping of genomic diversity in *Candida albicans*. Genome Res.

[b8] De Backer MD, Van Dijck P (2003). Progress in functional genomics approaches to antifungal drug target discovery. Trends Microbiol.

[b9] Dorer RK, Zhong S, Tallarico JA, Wong WH, Mitchison TJ, Murray AW (2005). A small-molecule inhibitor of Mps1 blocks the spindle-checkpoint response to a lack of tension on mitotic chromosomes. Curr Biol.

[b10] Enos AP, Morris NR (1990). Mutation of a gene that encodes a kinesin-like protein blocks nuclear division in *A. nidulans*. Cell.

[b11] Gerami-Nejad M, Berman J, Gale CA (2001). Cassettes for PCR-mediated construction of green, yellow, and cyan fluorescent protein fusions in *Candida albicans*. Yeast.

[b12] Giaever G, Chu AM, Ni L, Connelly C, Riles L, Veronneau S (2002). Functional profiling of the *Saccharomyces cerevisiae* genome. Nature.

[b13] Hagan I, Yanagida M (1990). Novel potential mitotic motor protein encoded by the fission yeast cut7+ gene. Nature.

[b14] Hazan I, Sepulveda-Becerra M, Liu H (2002). Hyphal elongation is regulated independently of cell cycle in *Candida albicans*. Mol Biol Cell.

[b15] Heald R (2000). Motor function in the mitotic spindle. Cell.

[b16] Heck MM, Pereira A, Pesavento P, Yannoni Y, Spradling AC, Goldstein LS (1993). The kinesin-like protein KLP61F is essential for mitosis in Drosophila. J Cell Biol.

[b17] Hoyt MA, Totis L, Roberts BT (1991). *S. cerevisiae* genes required for cell cycle arrest in response to loss of microtubule function. Cell.

[b18] Hoyt MA, He L, Loo KK, Saunders WS (1992). Two *Saccharomyces cerevisiae* kinesin-related gene products required for mitotic spindle assembly. J Cell Biol.

[b19] Jaspersen SL, Winey M (2004). The budding yeast spindle pole body: structure, duplication, and function. Annu Rev Cell Dev Biol.

[b20] Jones T, Federspiel NA, Chibana H, Dungan J, Kalman S, Magee BB (2004). The diploid genome sequence of *Candida albicans*. Proc Natl Acad Sci USA.

[b21] Jordan MA, Wilson L (2004). Microtubules as a target for anticancer drugs. Nat Rev Cancer.

[b22] Kapoor TM, Mitchison TJ (2001). Eg5 is static in bipolar spindles relative to tubulin: evidence for a static spindle matrix. J Cell Biol.

[b23] Kapoor TM, Mayer TU, Coughlin ML, Mitchison TJ (2000). Probing spindle assembly mechanisms with monastrol, a small molecule inhibitor of the mitotic kinesin, Eg5. J Cell Biol.

[b24] Li R, Murray AW (1991). Feedback control of mitosis in budding yeast. Cell.

[b25] Lila T, Renau TE, Wilson L, Philips J, Natsoulis G, Cope MJ, Watkins WJ, Buysse J (2003). Molecular basis for fungal selectivity of novel antimitotic compounds. Antimicrob Agents Chemother.

[b26] Lo HJ, Kohler JR, DiDomenico B, Loebenberg D, Cacciapuoti A, Fink GR (1997). Nonfilamentous *C. albicans* mutants are avirulent. Cell.

[b27] Luo L, Carson JD, Dhanak D, Jackson JR, Huang PS, Lee Y (2004). Mechanism of inhibition of human KSP by monastrol: insights from kinetic analysis and the effect of ionic strength on KSP inhibition. Biochemistry.

[b28] Maliga Z, Kapoor TM, Mitchison TJ (2002). Evidence that monastrol is an allosteric inhibitor of the mitotic kinesin Eg5. Chem Biol.

[b29] Mayer TU, Kapoor TM, Haggarty SJ, King RW, Schreiber SL, Mitchison TJ (1999). Small molecule inhibitor of mitotic spindle bipolarity identified in a phenotype-based screen. Science.

[b30] Michel S, Ushinsky S, Klebl B, Leberer E, Thomas D, Whiteway M, Morschhauser J (2002). Generation of conditional lethal *Candida albicans* mutants by inducible deletion of essential genes. Mol Microbiol.

[b31] Miglarese MR, Carlson RO (2006). Development of new cancer therapeutic agents targeting mitosis. Expert Opin Invest Drugs.

[b32] Moir DT, Shaw KJ, Hare RS, Vovis GF (1999). Genomics and antimicrobial drug discovery. Antimicrob Agents Chemother.

[b33] Mountain V, Simerly C, Howard L, Ando A, Schatten G, Compton DA (1999). The kinesin-related protein, HSET, opposes the activity of Eg5 and cross-links microtubules in the mammalian mitotic spindle. J Cell Biol.

[b34] O'Connell MJ, Meluh PB, Rose MD, Morris NR (1993). Suppression of the bimC4 mitotic spindle defect by deletion of klpA, a gene encoding a KAR3-related kinesin-like protein in *Aspergillus nidulans*. J Cell Biol.

[b35] Pidoux AL, LeDizet M, Cande WZ (1996). Fission yeast pkl1 is a kinesin-related protein involved in mitotic spindle function. Mol Biol Cell.

[b36] Reuss O, Vik A, Kolter R, Morschhauser J (2004). The SAT1 flipper, an optimized tool for gene disruption in *Candida albicans*. Gene.

[b37] Rice S, Lin AW, Safer D, Hart CL, Naber N, Carragher BO (1999). A structural change in the kinesin motor protein that drives motility. Nature.

[b38] Roof DM, Meluh PB, Rose MD (1992). Kinesin-related proteins required for assembly of the mitotic spindle. J Cell Biol.

[b39] Sablin EP, Kull FJ, Cooke R, Vale RD, Fletterick RJ (1996). Crystal structure of the motor domain of the kinesin-related motor ncd. Nature.

[b40] Saunders W, Lengyel V, Hoyt MA (1997). Mitotic spindle function in *Saccharomyces cerevisiae* requires a balance between different types of kinesin-related motors. Mol Biol Cell.

[b41] Saunders WS, Hoyt MA (1992). Kinesin-related proteins required for structural integrity of the mitotic spindle. Cell.

[b42] Saville SP, Lazzell AL, Monteagudo C, Lopez-Ribot JL (2003). Engineered control of cell morphology *in vivo* reveals distinct roles for yeast and filamentous forms of *Candida albicans* during infection. Eukaryot Cell.

[b43] Sawin KE, LeGuellec K, Philippe M, Mitchison TJ (1992). Mitotic spindle organization by a plus-end-directed microtubule motor. Nature.

[b44] Sharp DJ, McDonald KL, Brown HM, Matthies HJ, Walczak C, Vale RD (1999). The bipolar kinesin, KLP61F, cross-links microtubules within interpolar microtubule bundles of Drosophila embryonic mitotic spindles. J Cell Biol.

[b45] Sharp DJ, Brown HM, Kwon M, Rogers GC, Holland G, Scholey JM (2000). Functional coordination of three mitotic motors in Drosophila embryos. Mol Biol Cell.

[b46] Shelanski ML, Taylor EW (1967). Isolation of a protein subunit from microtubules. J Cell Biol.

[b47] Touitou I, Lhomond G, Pruliere G (2001). Boursin, a sea urchin bimC kinesin protein, plays a role in anaphase and cytokinesis. J Cell Sci.

[b48] Vale RD, Fletterick RJ (1997). The design plan of kinesin motors. Annu Rev Cell Dev Biol.

[b49] Vale RD, Milligan RA (2000). The way things move: looking under the hood of molecular motor proteins. Science.

[b50] Walczak CE, Vernos I, Mitchison TJ, Karsenti E, Heald R (1998). A model for the proposed roles of different microtubule-based motor proteins in establishing spindle bipolarity. Curr Biol.

[b51] Walsh C (2003). Antibiotics: Actions, Origins, Resistance.

[b52] Warner SL, Gray PJ, Von Hoff DD (2006). Tubulin-associated drug targets: aurora kinases, Polo-like kinases, and others. Semin Oncol.

[b53] Whelan WL, Magee PT (1981). Natural heterozygosity in *Candida albicans*. J Bacteriol.

[b54] Wills EA, Redinbo MR, Perfect JR, Del Poeta M (2000). New potential targets for antifungal development. Expert Opin Ther Targets.

[b55] Wilson RB, Davis D, Mitchell AP (1999). Rapid hypothesis testing with *Candida albicans* through gene disruption with short homology regions. J Bacteriol.

