# Supplementary material for: Effective killing of the human pathogen Candida albicans by a specific inhibitor of non-essential mitotic kinesin Kip1p
Source: Mol Microbiol. 2007 Jul 1;65(2):347–62. doi: 10.1111/j.1365-2958.2007.05787.x (PMC1976386; doi:10.1111/j.1365-2958.2007.05787.x)
Supplement: Fig. S1 — CaKIP1 encodes a non-essential member of the bimC kinesin family. Multiple sequence alignment of the bimC minimal motor domain from various species. Af = Aspergillus fumigatus; An = Aspergillus nidulans; Ca = Candida albicans; Cg = Candida glabrata; Sc= Saccharomyces cerevisiae; Hs = Homo sapiens; Sp= Schizosaccharomyces pombe. The conserved glycine required for ATP hydrolysis is underlined. [file mmi0065-0347-fs1.pdf]

```
                1                                                                                                 100
   AfBimC   (1) -TSIHVVVRCRGRNDREIKENSGVVVSTEGAKGK----------------TVELSMG---PNA-----------------VSNKAYTFDKVFSAAADQVT
   AnBimC   (1) DTSIHVVVRCRGRNEREVKENSGVVLQTEGVKGK----------------TVELSMG---PNA-----------------VSNKTYTFDKVFSAAADQIT
   ScKip1   (1) -SNIHVYVRCRSRNKREIEEKSSVVISTLGPQGK----------------EIILSNGSHQSYS-----------------SSKKTYQFDQVFGAESDQET
   CaKip1   (1) -SNIQVVVRCRGRNSQEIAAKSPIVVELTNDNYSITEPYITINQGYTRATATTTNNTTTTNSNNTAGATPNTSASSLDTNLKRTYKFDQIYGSQADQNL
   CgCin8   (1) -LNILVAVRCRGRNEREIKAKSSVVVDVPDNGVTNEV-------------SINTTDDVGIAAK-----------------MNSKTYTVDKVFGPSASQKL
   ScCin8   (1) -LNITVAVRCRGRNEREISMKSSVVVNVPDITGSKEI-------------SINTTGDTGITAQ-----------------MNAKRYTVDKVFGPGASQDL
    HsKSP   (1) GKNIQVVVRCRPFNLAERKASAHSIVECDPVRKEVSV-------------RTGGLADK----------------------SSRKTYTFDMVFGASTKQID
   SpCut7   (1) -TNINVVVRVRGRTDQEVRDNSSLAVSTSGAMGA----------------ELAIQSDP--SSM-----------------LVTKTYAFDKVFGPEADQLM
Consensus   (1)  TNINVVVRVRGRTDQEVRDNSSLAVSTSGAMGA                ELAIQSDP  SSM                 LVTKTYAFDKVFGPEADQLM

                101                                                                                               200
   AfBimC  (64) VYEDVVLPIVNEMLAGYNCTIFAYGQTGTGKTYTMSGDMTDTLGILSDN-------AGIIPRVLYSLFHKLEDT-----ESTVKCSFIELYNEELRDLLS
   AnBimC  (65) VYEDVVLPIVTEMLAGYNCTIFAYGQTGTGKTYTMSGDMTDTLGILSDN-------AGIIPRVLYSLFAKLADT-----ESTVKCSFIELYNEELRDLLS
   ScKip1  (67) VFNATAKNYIKEMLHGYNCTIFAYGQTGTGKTYTMSGDINILGDVQSTDNLLLGEHAGIIPRVLVDLFKELSSLNK---EYSVKISFLELYNENLKDLLS
   CaKip1 (100) IYSQIAHPLLLDFLRGINVTVLAYGQTGTGKTYTMSGMDN--NNKLDEN----SDIAGIIPRLLGELFQNLSSNATSKNDYMVKISYLEIYNEELIDLLS
   CgCin8  (70) VYEEIAEPLFQDFIKGYNCTILVYGMTSTGKTYTMTGDEKLHNGELGDA-------AGIIPRVLFELFDTLEANKD---DYLVKCSFVELYNEELKDLLD
   ScCin8  (70) IFDEVAGPLFQDFIKGYNCTVLVYGMTSTGKTYTMTGDEKLYNGELSDA-------AGIIPRVLLKLFDTLELQQN---DYVVKCSFIELYNEELKDLLD
    HsKSP  (66) VYRSVVCPILDEVIMGYNCTIFAYGQTGTGKTFTMEGERSPNEEYTWEEDP----LAGIIPRTLHQIFEKLTDNGT---EFSVKVSLLEIYNEELFDLLN
   SpCut7  (65) LFENSVAPMLEQVLNGYNCTIFAYGQTGTGKTYTMSGDLSDSDGILSEG-------AGLIPRALYQLFSSLDNSNQ---EYAVKCSYYELYNEEIRDLLV
Consensus (101) LFENSVAPMLEQVLNGYNCTIFAYGQTGTGKTYTMSGDLSDSDGILSEG       AGLIPRALYQLFSSLDNSNQ   EYAVKCSYYELYNEEIRDLLV

                201                                                                                               300
   AfBimC (152) AEEN----------PK--LKIFENEKKG------------------------------------------------------------------------
   AnBimC (153) AEEN----------PK--LKIYDNEQKK------------------------------------------------------------------------
   ScKip1 (164) DSEDDDPAVN---DPKRQIRIFDNNN--------------------------------------------------------------------------
   CaKip1 (194) NNNSNNR----------KLRIHEKTNAN------------------------------------------------------------------------
   CgCin8 (160) STNTATN------SDNKKLRIFDSNVNG--TSASGSSSRSSSRNNSPRSAPDNSRAQMLRRKLGRHNTTGNSKISNNNHNKFSRFKQTSQESTRAHASNN
   ScCin8 (160) SNSNGSSNTGFDGQFMKKLRIFDSSTANNTTSNSASSSRSNSRNSSPRSLNDLTPKAALLRKRLRTKSLPNTIKQQYQQQQAVNSRNNS-------SSNS
    HsKSP (159) PSSDVSER----------LQMFDDPRN-------------------------------------------------------------------------
   SpCut7 (155) SEELRKP-----------ARVFEDTS--------------------------------------------------------------------------
Consensus (201) SEELRKP           ARVFEDTS

                301                                                                                               400
   AfBimC (168) --------------------T-----SGSTLVQGMEETWIDSASAGIKLLQLGSHKRQVAATKCNDLSSRSHTIFTITVYTKRTTENGD-----------
   AnBimC (169) --------------------G-----HMSTLVQGMEETYIDSATAGIKLLQQGSHKRQVAATKCNDLSSRSHTVFTITVNIKRTTESGE-----------
   ScKip1 (187) --------------------N-----NSSIMVKGMQEIFINSAHEGLNLLMQGSLKRKVAATKCNDLSSRSHTVFTITTNIVEQDSKDHGQ--------N
   CaKip1 (212) -------------------------KVKSIGIQNLTEYTINNYNQGIKYLKMGLQKKKTSSTNLNETSSRSHTIFSVQLYQKISSSSSSSSE----NDNE
   CgCin8 (252) HQNVHIPNNNTNNTNQQQS---PIDQSASIYIQNLEEFHITSAMEGLQLLQKGLKQRQVASTKMNDFSSRSHSIFTITLYKEQNG---------------
   ScCin8 (253) GSTTNNASSNTNTNNGQRSSMAPNDQTNGIYIQNLQEFHITNAMEGLNLLQKGLKHRQVASTKMNDFSSRSHTIFTITLYKKHQD---------------
    HsKSP (176) --------------------------KRGVIIKGLEEITVHNKDEVYQILEKGAAKRTTAATLMNAYSSRSHSVFSVTIHMKETTIDG------------
   SpCut7 (170) --------------------R-----RGNVVITGIEESYIKNAGDGLRLLREGSHRRQVAATKCNDLSSRSHSIFTITLHRKVSSGMTDETNSLTINNNS
Consensus (301)                     R     RGNVVITGIEESYIKNAGDGLRLLREGSHRRQVAATKCNDLSSRSHSIFTITLHRKVSSGMTDETNSLTINNNS
```

```
                 401                                                                                                500
   AfBimC  (232) -DYISSGKLNLVDLAGSENIQRSGAENKRATEAGLINKSLLTLGRVINALVDK-SPHIPYRESKLTRLLQDSLGGRTKTCIIATISPSRSNLEETISTLD
   AnBimC  (233) -EYVCPGKLNLVDLAGSENIGRSGAENKRATEAGLINKSLLTLGRVINALVDK-SQHIPYRESKLTRLLQDSLGGRTKTCIIATMSPARSNLEETISTLD
   ScKip1  (254) KNFVKIGKLNLVDLAGSENINRSGAENKRAQEAGLINKSLLTLGRVINALVDH-SNHIPYRESKLTRLLQDSLGGMTKTCIIATISPAKISMEETASTLE
   CaKip1  (283) ESVYRISKMNLVDLAGSENISRSGS---IVKEAGGINQSLLTLGRVINSLNEKKLLHIPYRESKLTHILQDSLGGNTKTTLIATISPAQVNLLETCSTLD
   CgCin8  (334) -ELFRVSKMNLVDLAGSENISRSGAMNQRAKEAGSINQSLLTLGRVINSLADK-SEHIPFRESKLTRLLQDSLGGNTKQHNCHYIS-CKMTSEETCSTLE
   ScCin8  (338) -ELFRISKMNLVDLAGSENINRSGALNQRAKEAGSINQSLLTLGRVINALVDK-SGHIPFRESKLTRLLQDSLGGNTKTALIATISPAKVTSEETCSTLE
    HsKSP  (238) EELVKIGKLNLVDLAGSENIGRSGAVDKRAREAGNINQSLLTLGRVITALVER-TPHVPYRESKLTRILQDSLGGRTRTSIIATISPASLNLEETLSTLE
   SpCut7  (245) DDLLRASKLHMVDLAGSENIGRSGAENKRARETGMINQSLLTLGRVINALVEK-AHHIPYRESKLTRLLQDSLGGKTKTSMIVTVSSTNTNLEETISTLE
Consensus  (401) DDLLRASKLHMVDLAGSENIGRSGAENKRARETGMINQSLLTLGRVINALVEK AHHIPYRESKLTRLLQDSLGGKTKTSMIVTVSSTNTNLEETISTLE

                 501       513
   AfBimC  (330) YAFRAKNIRNKPQ
   AnBimC  (331) YAFRAKNIRNKPQ
   ScKip1  (353) YATRAKSIKNTPQ
   CaKip1  (380) YASKAKNIKNAPM
   CgCin8  (431) YASKAKNIKNKPQ
   ScCin8  (436) YASKAKNIKNKPQ
    HsKSP  (337) YAHRAKNILNKPE
   SpCut7  (344) YAARAKSIRNKPQ
Consensus  (501) YAARAKSIRNKPQ
```

Supplementary Figure 1. *CaKIP1* encodes a non-essential member of the bimC kinesin family. Multiple sequence alignment of the bimC minimal motor domain from various species. Af = *Aspergillus fumigatus*; An = *Aspergillus nidulans*; Ca = *Candida albicans*; Cg = *Candida glabrata*; Sc = *Saccharomyces cerevisiae*; Hs = *Homo sapiens*; Sp = *Schizosaccharomyces pombe*. The conserved glycine required for ATP hydrolysis is underlined.
